# Negative regulation of DAB2IP by Akt and SCF*^Fbw7^* pathways

**DOI:** 10.18632/oncotarget.1939

**Published:** 2014-05-01

**Authors:** Xiangping Dai, Brian J. North, Hiroyuki Inuzuka

**Affiliations:** ^1^ Department of Pathology, Beth Israel Deaconess Medical Center, Harvard Medical School, Boston, MA

**Keywords:** DAB2IP, Akt, Fbw7, degradation, cancer, phosphorylation, ubiquitination

## Abstract

Deletion of ovarian carcinoma 2/disabled homolog 2 (DOC-2/DAB2) interacting protein (DAB2IP), is a tumor suppressor that serves as a scaffold protein involved in coordinately regulating cell proliferation, survival and apoptotic pathways. DAB2IP is epigenetically down-regulated in a variety of tumors through the action of the histone methyltransferase EZH2. Although DAB2IP is transcriptionally down-regulated in a variety of tumors, it remains unclear if other mechanisms contribute to functional inactivation of DAB2IP. Here we demonstrate that DAB2IP can be functionally down-regulated by two independent mechanisms. First, we identified that Akt1 can phosphorylate DAB2IP on S847, which regulates the interaction between DAB2IP and its effector molecules H-Ras and TRAF2. Second, we demonstrated that DAB2IP can be degraded in part through ubiquitin-proteasome pathway by SCF^Fbw7^. DAB2IP harbors two Fbw7 phosho-degron motifs, which can be regulated by the kinase, CK1δ. Our data hence indicate that in addition to epigenetic down-regulation, two additional pathways can functional inactivate DAB2IP. Given that DAB2IP has previously been identified to possess direct causal role in tumorigenesis and metastasis, our data indicate that a variety of pathways may pass through DAB2IP to govern cancer development, and therefore highlight DAB2IP agonists as potential therapeutic approaches for future anti-cancer drug development.

## INTRODUCTION

Tumor metastasis is a substantial setback encountered during clinical anti-cancer treatments, leading to increased mortality in cancer patients [1]. It has been well established that for many types of human cancers, tumor cells acquire the capability to metastasize to distant organs that ultimately result in organ failure and death [1, 2]. Therefore, elucidating the underlying molecular mechanisms that drive tumor growth and metastasis will provide further impetus for the development of more effective therapies, in part by eliminating metastatic cancer cells. Although the mechanisms remain largely unknown, overexpression of certain oncoproteins [3] or down-regulation of tumor suppressor proteins [4] have been demonstrated to play important roles in the process of tumor growth and metastasis. Deletion of ovarian carcinoma 2/disabled homolog 2 (DOC-2/DAB2) interacting protein (DAB2IP), has been described as a tumor suppressor in various types of cancer [5-9]. Furthermore, loss of DAB2IP expression during tumorigenesis is associated with poor prognosis and increased tumor metastasis [6, 8-11].

DAB2IP is often down-regulated by epigenetic modification in multiple aggressive cancers. In prostate cancer, DAB2IP expression was shown to be repressed by promoter methylation and histone modification, primarily through the action of the histone methyltransferase EZH2 [12, 13], whereas in breast cancer [6], lung cancer [8], and gastrointestinal tumors [14], aberrant promoter hypermethylation was shown to down-regulate DAB2IP. Moreover, it was shown in prostate cancer that down-regulation of DAB2IP expression results in resistance to ionizing radiation [15], which initiates epithelial-to-mesenchymal transition [11] and promotes tumor growth and metastasis [16]. Mechanistically, DAB2IP is involved in TNFα-induced apoptosis in prostate cancer cells by suppressing the ASK1-JNK and PI3K-Akt pathway [11], and in endothelial cells via the ASK1-JNK pathway [17].

DAB2IP, located at chromosome 9q33.1-q33.3, is a member of the Ras-GTPase activating protein family (RAS GAP) that inactivates Ras largely by promoting conversion of GTP into GDP [18]. Functionally, DAB2IP serves as a scaffold protein involved in coordinately regulating cell proliferation, survival and apoptotic pathways [19]. Through interaction with various factors, DAB2IP can modulate the activities of various pathways including Ras-Raf-ERK, ASK1-JNK, and PI3K-Akt, through which loss of DAB2IP can deregulate survival and apoptosis pathways, leading to tumor development. Specifically, DAB2IP inhibits the RAS pathway by directly binding to and inactivating H-Ras and R-Ras through its Ras GTPase activity [18], regulates the ASK1 pathway by blocking interaction of ASK1 with its inhibitor 14-3-3 [17], and binds to and inactivates the Akt kinase [19].

Prostate cancer is largely curable if diagnosed early in disease progression, however, there are no effective therapies for metastatic disease. Recent studies have identified DAB2IP as a regulator of metastatic prostate cancer, and is one of the few genes with a direct causal role in driving prostate cancer metastasis. DAB2IP has roles in both tumor initiation and metastasis, whereby DAB2IP controls primary tumor growth through activating Ras and drives metastasis through controlling NF-κB through regulation of TRAF2 [16]. Although DAB2IP is often epigenetically down regulated in a variety of cancers through EZH2 activation, we set out to determine if DAB2IP can also be functionally inactivated through signaling or degradation pathways. To this end, we have identified that DAB2IP is functionally inactivated by Akt-mediated phosphorylation, controlling DAB2IP activity on both the Ras and TRAF2 downstream effector molecules. In addition, we have identified that DAB2IP protein levels can be controlled by Fbw7-mediated ubiquitination and subsequent degradation.

## RESULTS

### DAB2IP is phosphorylated by Akt1

**Figure 1:**
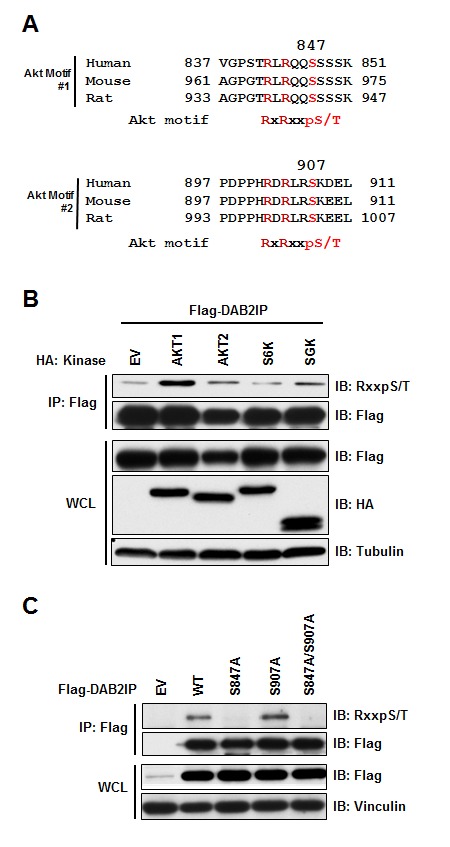
DAB2IP is phosphorylated by Akt1 (A) Alignment of human, mouse, and rat DAB2IP sequences surrounding putative Akt substrate motif. (B) 293T cells transfected with FLAG-DAB2IP with vector alone, Akt1, Akt2, S6K, or SGK were immunoprecipitated with anti-FLAG, and western blotted with antibodies against RxxpS/T, FLAG, HA, and Tubulin. (C) 293T cells transfected with wild-type, S847A, S907A and S847A/S907A FLAG-DAB2IP were immunoprecipitated with anti-FLAG, and western blotted with antibodies against RxxpS/T, FLAG, and Viniculin.

Previous studies demonstrated that DAB2IP is epigenetically down regulated in many tumors through the action of EZH2 or promoter methylation [6, 8, 12-14]. We set out to determine if there are additional mechanisms functionally regulating DAB2IP. Scanning the DAB2IP protein sequence, we identified two consensus Akt sites (RxRxxpS/T) located in the carboxy terminus of DAB2IP at Serine-847 and Serine-907 (Figure 1A). To test if this consensus sequence could serve as a *bona fide* phosphorylation site in DAB2IP, we tested if any Akt or similar kinases (Akt1, Akt2, SGK and ribosomal S6 kinase (S6K)) were able to phosphorylate DAB2IP. Using a phospho-Akt substrate specific antibody, we found that only Akt1 expression led to increased phosphorylation of DAB2IP (Figure 1B). By mutating each phosphorylation site within the two consensus Akt motifs, we found that S847 was primarily phosphorylated by Akt1 (Figure 1C). These data indicate that Akt1 can phosphorylate DAB2IP in the carboxy terminus at S847.

### Phosphorylated DAB2IP blocks interaction with H-Ras and TRAF2

**Figure 2:**
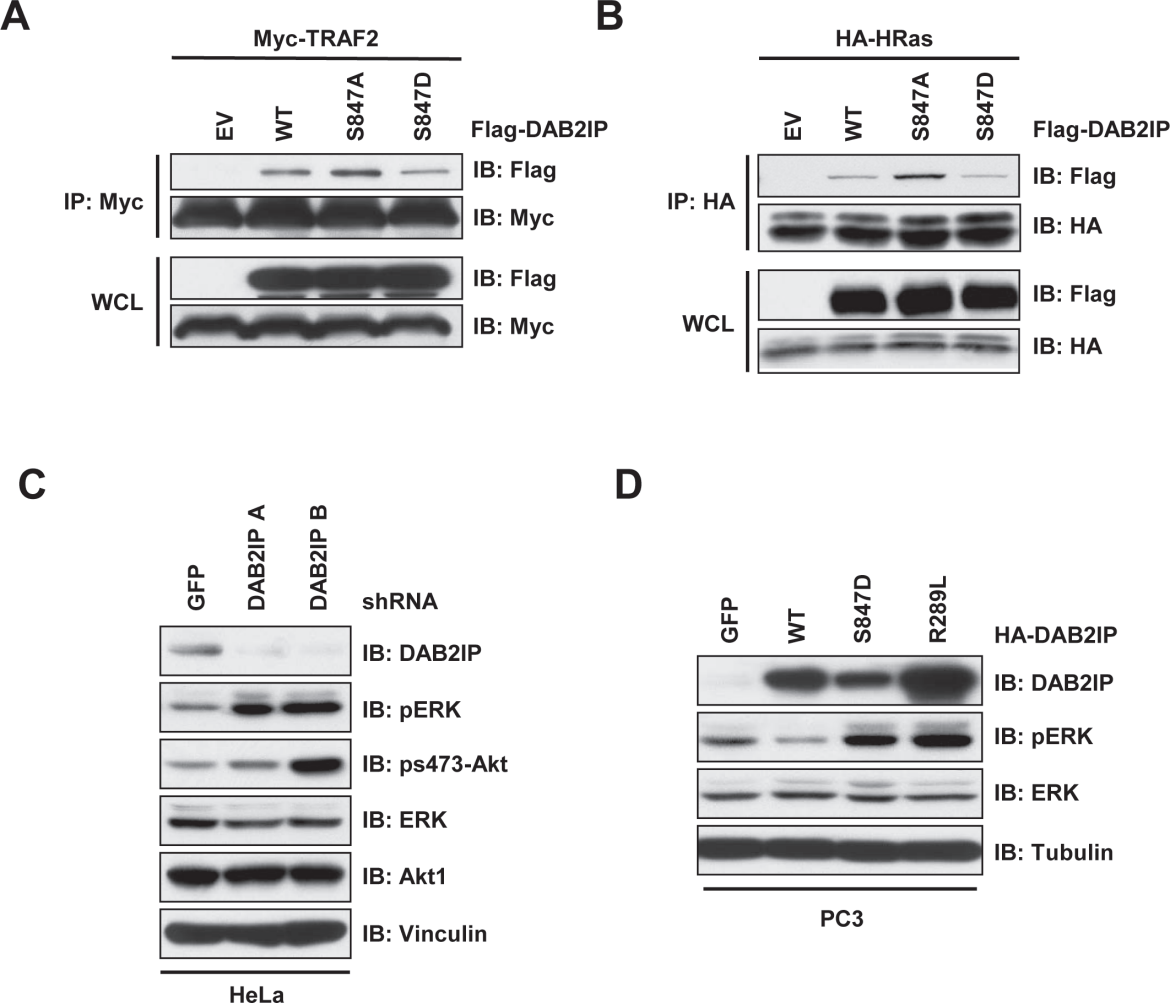
Phosphorylation at S847 controls DAB2IP function (A) 293T cells transfected HA-H-Ras with vector alone, wild-type and S847A and S847D FLAG-DAB2IP were immunoprecipitated with anti-HA, and western blotted with antibodies against FLAG and HA. (B) 293T cells transfected Myc-TRAF2 with vector alone, wild-type and S847A and S847D FLAG-DAB2IP were immunoprecipitated with anti-Myc, and western blotted with antibodies against FLAG and Myc. (C) PC3 cells transduced with lentivirus expressing GFP, wild-type, S847D or R289L HA-DAB2IP were western blotted with antibodies against DAB2IP, pERK, total ERK, and Tubulin. (D) HeLa cells were infected with virus expressing shRNA against GFP or DAB2IP. Following selection of infected cells, lysates were western blotted with antibodies against DAB2IP, pERK, total ERK, Akt pS475, total Akt, and Vinculin.

Loss of DAB2IP was shown to trigger RAS, ERK and Akt activation [16], and interact with TRAF2 via its C-terminal domain [20]. Interestingly, our identified Akt1 phosphorylation site lies in the C-terminus of the proline-rich interaction domain in DAB2IP that is involved in binding TRAF2 and ASK1. To test if phosphorylation at S847 by Akt1 also influences the association of DAB2IP and TRAF2, we tested the interaction of phospho-mimetic (S847D) and non-phosphorylatable (S847A) mutants with TRAF2. Notably, we found that the DAB2IP S847A mutant bound more efficiently while the DAB2IP S847D mutant had reduced binding to TRAF2 (Figure 2A). Likewise, we found that the ability of DAB2IP to bind to Ras was also affected by the phosphorylation status of DAB2IP as the interaction between DAB2IP and Ras is regulated by the phosphorylation status at S847, with the non phosphorylation substitution (S847A) having increased interaction, while a phosphomimetic substitution (S847D) showing reduced binding (Figure 2B). These results indicate that the ability of the scaffold protein DAB2IP to interact with TRAF2 and Ras is controlled in part through Akt1-dependent phosphorylation in the C-terminus of DAB2IP.

In addition to TRAF2, DAB2IP has been shown to regulate the RAS-ERK signaling pathway, where depletion of DAB2IP leads to MAPK pathway activation (Figure 2C and [16, 19]). To test if phosphorylation at the Akt site of DAB2IP is important for its ability to control MAPK pathway activation, we assessed the MAPK activation in PC3 cells, which have limited expression of DAB2IP. We found that expression of wild-type DAB2IP resulted in lower MAPK activation as measured by phosphorylation of ERK (Figure 2D). Expression of the phospho-mimetic mutant (S847D) of DAB2IP resulted in an increase in MAPK activation (Figure 2D). Induced MAPK activity that we observed with phosphorylation at S847D was similar to what was observed for a catalytically inactive RasGAP mutant of DAB2IP (R289L), suggesting that the regulation of DAB2IP binding to Ras was important for DAB2IP to control MAPK activity. Therefore, our results indicate that phosphorylation at S847 of DAB2IP is important for its downstream effector functions, and thus regulation of the phosphorylation status at S847 is important for the tumor suppressor roles of DAB2IP.

### DAB2IP interacts with Cullin-Ring E3 ligases

Given that DAB2IP is a potent tumor suppressor, and is down-regulated in a variety of human tumors, we intend to determine if DAB2IP is actively regulated by proteasome-mediated degradation in addition to being functionally regulated by phosphorylation. To this end, the ubiquitin proteasome system (UPS) plays an important role in the timely regulation of key cellular proteins and thereby controlling many cellular processes including cell signaling and cell cycle regulation [21]. Dysfunction of the UPS is involved in the development of many diseases including cancer [22, 23].

**Figure 3:**
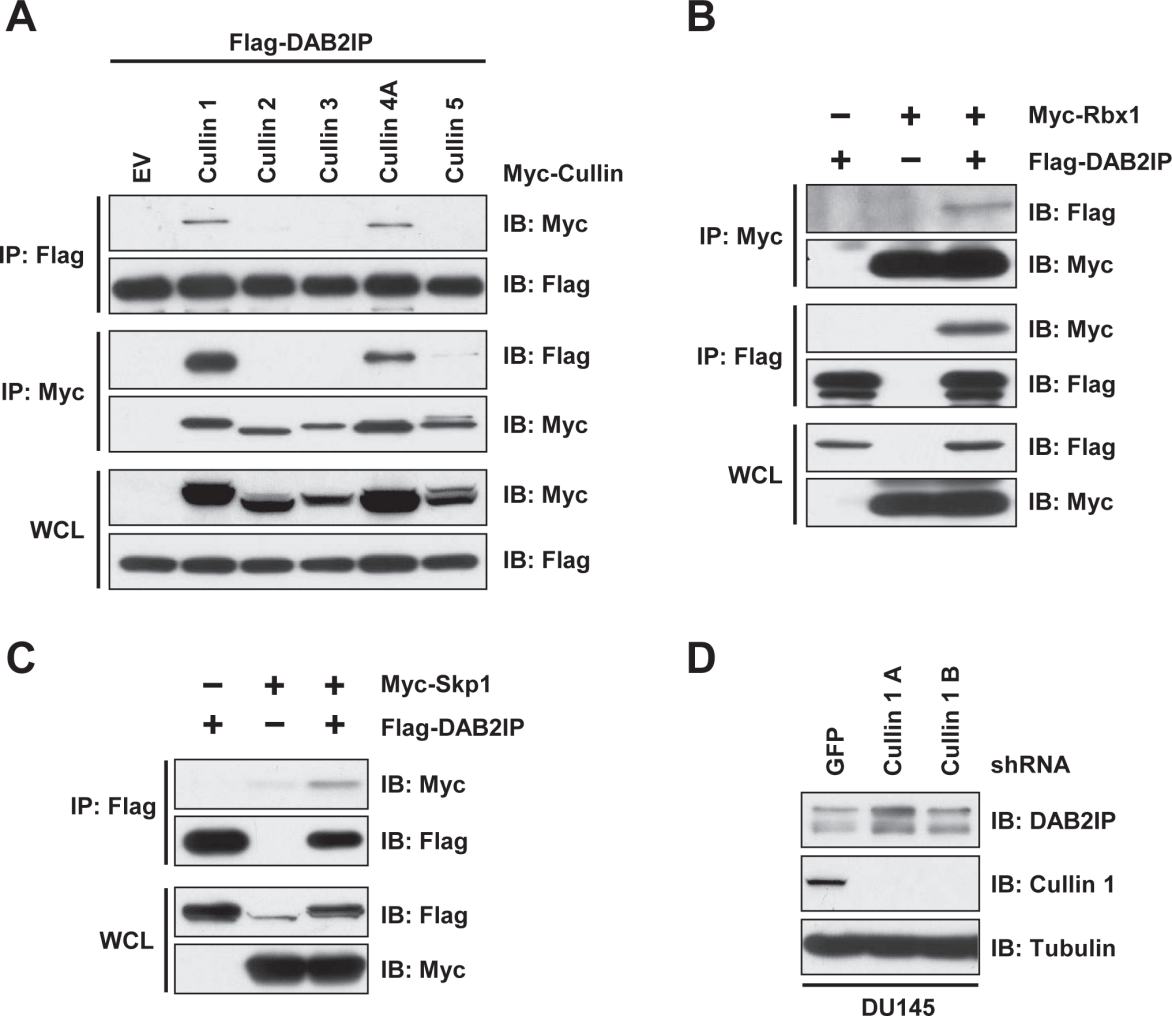
DAB2IP stability is controlled by Cullin-Ring mediated degradation (A) 293T cells transfected FLAG-DAB2IP with vector alone, or Myc-tagged Cullin 1-5 were immunoprecipitated with anti-FLAG or anti-Myc, and western blotted with antibodies against FLAG and Myc. (B) 293T cells transfected FLAG-DAB2IP with vector alone or Myc-Rbx1 were immunoprecipitated with anti-FLAG or anti-Myc, and western blotted with antibodies against FLAG and Myc. (C) 293T cells transfected FLAG-DAB2IP with vector alone, or Myc-Skp1 were immunoprecipitated with anti-FLAG or anti-Myc, and western blotted with antibodies against FLAG and Myc. (D) DU145 cells were infected with virus expressing shRNA against GFP or 2 independent shRNAs to Cullin 1. Following selection of infected cells, lysates were western blotted with antibodies against DAB2IP, Cullin 1, and Tubulin.

It is well established that Cullin-Ring complexes are the largest family of E3 ligases. Therefore, we determined whether a specific Cullin-Ring E3 ligase interacts with DAB2IP. We found that DAB2IP interacted with Cullin-1 and Cullin-4A, while it did not interact with Cullin-2, Cullin-3, or Cullin-5 (Figure 3A). Cullin-1 based E3 ligases are the most well studied Cullin-Ring complexes, and are named SCF complexes due to the presence of the components Skp1, Cullin-1 and an associated F-box protein that directs substrate recognition. Consistent with an interaction between Cullin-1 and DAB2IP, we found that DAB2IP interacted with Rbx1 and Skp1, common complex component of Cullin-1 based SCF complexes (Figure 3B and 3C). Furthermore, depletion of cells with Cullin-1 shRNA resulted in an increase in DAB2IP protein abundance (Figure 3D).

### SCF**^Fbw7^**regulates DAB2IP stability

**Figure 4:**
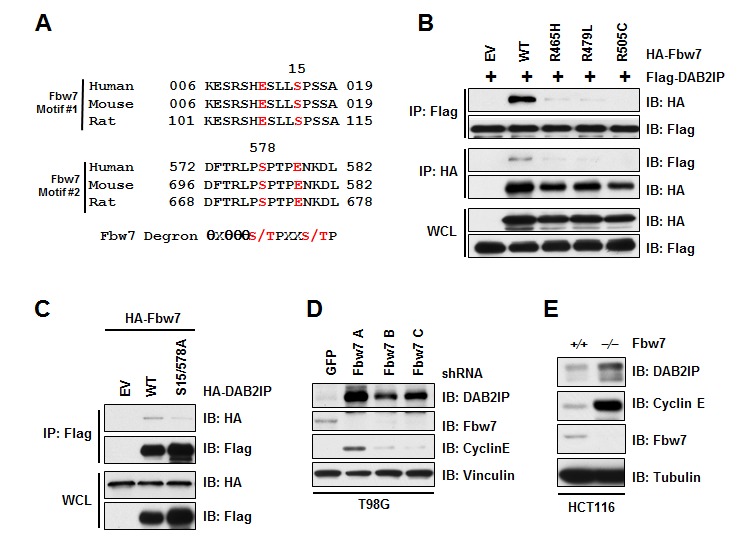
Fbw7 interacts with and regulates DAB2IP stability (A) Alignment of human, mouse, and rat DAB2IP sequences surrounding putative Fbw7 degron motif. (B) 293T cells transfected with FLAG-DAB2IP and vector alone, wild-type, R465H, R479L, or R505C HA-Fbw7 were immunoprecipitated with either anti-FLAG or anti-HA, and western blotted with antibodies against FLAG and HA. (C) 293T cells transfected HA-Fbw7 with vector alone, wild-type or S15/578A FLAG-DAB2IP were immunoprecipitated with anti-FLAG, and western blotted with antibodies against FLAG and HA. (D) T98G cells were infected with virus expressing shRNA against GFP or Fbw7. Following selection of infected cells, lysates were western blotted with antibodies against DAB2IP, Fbw7, Cyclin E, and Vinculin. (E) Wild-type and Fbw7 knockout HCT116 cells were western blotted with antibodies against DAB2IP, Fbw7, Cyclin E, and Tubulin.

The human genome harbors 69 F-Box proteins, where β-TRCP and Fbw7 are the most well-established substrate recognition components of the SCF complex, and dysregulation of these F-box proteins can contribute to cancer development [24-26]. Scanning the DAB2IP protein sequence, we identified two potential phospho-degron sequences (S15 and S578) that share partial homology to the consensus Fbw7 substrate recognition motif (Figure 4A). We found that Fbw7 interacted with DAB2IP (Figure 4B), indicating a role for Fbw7 as the F-Box protein involved in recruiting DAB2IP to the SCF complex. Fbw7 is frequently mutated in T-ALL, and plays a critical role in T-ALL cancer development [27], where common mutations in Fbw7 found in T-ALL abrogate interaction between Fbw7 and substrate proteins. Consistent with DAB2IP being a substrate of Fbw7, we found that three T-ALL associated mutations in Fbw7 (R465H, R479L, and R505C) no longer interacted of Fbw7 with DAB2IP (Figure 4B), providing further evidence that DAB2IP interaction with Fbw7 is through the substrate recognition domain of Fbw7. We next tested if mutation of the Fbw7 phospho-degron motifs in DAB2IP resulted in loss of biding between these two factors. Mutation of Serine-15 and Serine 578 in DAB2IP resulted in loss of binding between DAB2IP and Fbw7 (Figure 4C). These results combined suggest that the substrate recognition F-Box motif in Fbw7 binds to the Fbw7 phospho-degron motif in DAB2IP.

With DAB2IP interacting with SCF^Fbw7^, we wanted to next determine if this Cullin-Ring E3 ligase controls the stability of DAB2IP. To test this, we depleted Fbw7 and assessed protein abundance of DAB2IP. We observed that knockdown of Fbw7 resulted in an increase in DAB2IP protein (Figure 4D). Additionally, deletion of Fbw7 in HCT116 cells also led to an increase in DAB2IP protein abundance (Figure 4E). Our results indicate that Fbw7 promotes the degradation of DAB2IP through recruitment to SCF^Fbw7^ complex for degradation.

### Phosphorylation-dependent interaction and degradation of DAB2IP

**Figure 5:**
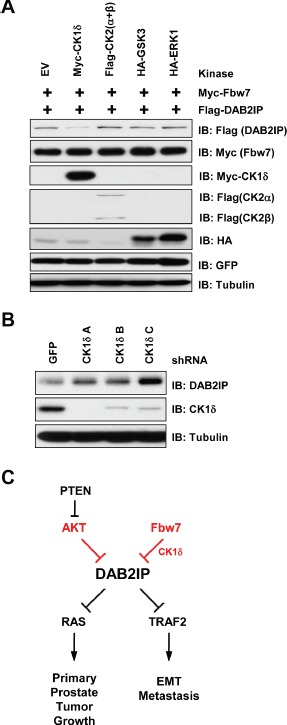
CK1δ regulates phosphorylation of the DAB2IP degron motif (A) 293T cells transfected with GFP, FLAG-DAB2IP and HA-Fbw7 with vector alone, CK1δ, CK2 (α and β), GSK3, or ERK1 were western blotted with antibodies against FLAG, GFP, and Tubulin. (B) 293T cells were infected with virus expressing shRNA against GFP or CK1δ. Following selection of infected cells, lysates were western blotted with antibodies against DAB2IP, CK1δ, and Tubulin. (C) Schematic depicting regulation of DAB2IP by Akt and Fbw7 pathways.

Fbw7-mediated degradation often depends on the presence of a phospho-degron motif in the target protein. Mutation of both of these serine residues led to a decrease in binding between Fbw7 and DAB2IP (Figure 4C), indicating that one, or both, of these motifs are important for interaction between DAB2IP and Fbw7. To determine which kinase is involved in the regulation of phosphorylation within DAB2IP degron motifs, we tested whether CK1α/β, CK1δ, GSK3, or ERK1 could alter protein abundance of DAB2IP. We found that overexpression of CK1δ led to a decrease in DAB2IP (Figure 5A). Furthermore, depletion of CK1δ resulted in an increase in DAB2IP protein abundance (Figure 5B). Our data is consistent with the notion that degradation of DAB2IP by SCF^Fbw7^ requires phosphorylation within the degron motif in DAB2IP, which is possibly carried out by CK1δ.

## DISCUSSION

DAB2IP is an emerging tumor suppressor, which is often epigenetically down-regulated in cancer. Given a direct causal role of DAB2IP for regulating cancer metastasis, we hypothesize that DAB2IP could be targeted for functional inactivation through additional posttranslational mechanisms. Here we identified two independent pathways that can negatively regulate DAB2IP function; inactivation through Akt mediated phosphorylation and degradation through SCF^Fbw7^-mediated ubiquitination and degradation.

Although DAB2IP is often epigenetically down regulated by EZH2 activation in many tumors, we defined a novel pathway of functionally inactivating DAB2IP in prostate cancer cells through Akt activity. We identified an Akt consensus sequence in DAB2IP, and demonstrated that this site is a possible target of Akt. Furthermore, we have found that phosphorylation of this site directly affects DAB2IP association with Ras and TRAF proteins, implicating this modification in controlling DAB2IP regulation of downstream effector pathways. This is of particular interest as Akt is negatively regulated by PTEN, suggesting that the loss of PTEN in prostate cancer could possibly inactivate DAB2IP through Akt activation, thereby perhaps leading to greater prevalence of prostate cancer metastasis (Figure 5C)

Recent results have shown that DAB2IP can suppress the Akt pathway through direct interaction. Our results suggest that, in addition to Akt inactivating DAB2IP, DAB2IP can control of Akt can lead to a positive feedback loop. Where increased Akt activity will further induce itself through negative regulation of DAB2IP. This feedback loop therefore leads to even greater Akt activity in cell, which are also primed to undergo metastasis through additional downstream pathways dysregulated with inhibition of DAB2IP. Therefore, targeting this feedback loop through increasing DAB2IP protein abundance or function could be a unique therapeutic possibility.

In addition to functional inactivation of DAB2IP by Akt, we identified that DAB2IP is also targeted for proteasome-mediated degradation driven by the SCF^Fbw7^ E3 ubiquitin ligase. To this end, we have identified that the SCF^Fbw7^ E3 ubiquitin ligase interacts with, and promotes the degradation of, DAB2IP. Furthermore, we have identified a phospho-degron motif within DAB2IP that is commonly utilized by Fbw7 for substrate recruitment to the SCF complex. We have identified CK1δ as a potential upstream modifying kinase regulating the phosphorylation of this Fbw7 phospho-degron motif within DAB2IP. Overexpression and knockdown of CK1δ leads to the loss and stabilization of DAB2IP, respectively, suggesting a direct role for CK1δ in mediating the degradation of DAB2IP by Fbw7. Identifying key pathways regulating degradation of DAB2IP could prove to be a potential therapeutic target by restoring DAB2IP protein abundance through inhibition of its degradation.

Recent studies have indicated that DAB2IP down-regulation has implications in resistance to ionizing radiation [15], initiation of the epithelial-to-mesenchymal transition [11] and promotes tumor growth and metastasis [16]. Here, by identifying that the protein stability of DAB2IP is regulated by the SCF^Fbw7^ E3 ubiquitin ligase, suggests that utilizing inhibitors of this E3 ligase complex could serve as a mechanism to block these processes by protecting DAB2IP protein levels in cellular contexts where DAB2IP expression is suppressed.

Interestingly, Fbw7 is often referred to as a tumor suppressor for its roles in regulating the degradation of potent oncogenes such as Cyclin E, c-Myc, c-Jun, Mcl-1, mTOR and Notch-1 [28]. Here we find that Fbw7 is involved in the degradation of a tumor suppressor. However, this has also recently been the case for another RasGAP molecules, neurofibromin (or NF1), which is also a known tumor suppressor [29]. The role of FBW7 regulating two known tumor suppressors, both of which are RasGAPs, for degradation, suggest a potential unifying role of DAB2IP and NF1 being under the control of Fbw7-mediated degradation which requires further studies of these pathways and their stability control by Fbw7 to elucidate the implications in cancer development and progression.

Given a causal role for DAB2IP in driving progression of prostate cancer towards metastasis, and the prevalence of PTEN loss early in prostate cancer development, understanding how DAB2IP is regulated is of critical importance. In summary, our results identify two new pathways leading to the functional inactivation of the tumor suppressor DAB2IP through Akt-mediated phosphorylation and SCF^Fbw7^-mediated degradation. Our work further suggest that in part by restoring DAB2IP expression to suppress cancer cell growth, proliferation and metastasis, Akt inhibitors or Fbw7/CKI inhibitors may be beneficial in treating various types of human cancers, particularly the metastatic cancers that are associated with poor survival rates.

## MATERIALS AND METHODS

### Cell Culture

HeLa, 293T, HCT116, and T98G cells were cultured in DMEM medium (Life Technologies, CA) supplemented with 10% FBS, penicillin and streptomycin. The cell lines DU145 and PC3 were cultured in RPMI 1640 medium with 10% FBS and antibiotics.

### Plasmids

Myc-Cullin 1, Myc-Cullin 2, Myc-Cullin 3, Myc-Cullin 4A, and Myc-Cullin 5 constructs were kind gifts from J. DeCaprio (Dana-Farber Cancer Institute, Boston, MA). HA-Fbw7 was a kind gift form Dr. Keiichi I Nakayama (Kyushu University, Fukuoka, Japan). ERK1 plasmid was a kind gift from Dr. John Blenis (Harvard Medical School, Boston, MA). HA-Ras plasmid was a kind gift from Dr. Lewis Cantly (Weill Cornell Medical College, New York, NY). shRNA CK1δ was a kind gift from Dr. William Hahn (Dana-Farber Cancer Institute). Myc-TRAF2, CK1δ, CK1α and CK1βδ where kind gifts from Dr. Wade Harper (Harvard Medical School). The following plasmids were previously described: Akt1, Akt2 and S6K [30]; SGK [31]; Myc-Rbx1, Myc-Skp1, GSK3, shRNA Cullin-1 and shRNA Fbw7 [32]; FLAG-DAB2IP and shRNA DAB2IP [16].

Cell transfection and viral transduction procedures

For cell transfection, 5×10^5^ HeLa or 293T cells were seeded in 100-mm plates and transfected using Lipofectamine (Invitrogen) in OptiMEM medium (Invitrogen) for 48 hours according to the manufacturer's instructions. For viral transduction experiments, 6×10^5^ HEK 293T cells were seeded in 60-mm dishes and cotransfected the next day with each lentivirus or retrovirus vector, along with helper plasmids (i.e., gag-pol and VSV-G were used for lentiviral infections). Media with progeny virus from transfected cells was collected every 24 h for 2 d, and then filtered with 0.45-μm filters (Millipore). After infection, the cells were selected with 1 μg/ml puromycin (Sigma-Aldrich) for 72 hours to eliminate the uninfected cells before collecting the whole cell lysates (WCLs) for the subsequent biochemical assays. Knockdown or overexpression in the transduced cells was confirmed by western blot analysis.

### Antibodies and Reagents

The following antibodies were used for this study. RxxpS/T (9614), pERK (4370), ERK (4905), pS473-Akt (4501), Akt (4691) and Anti-Cullin 1 (4995) were from Cell Signaling Technology. Cyclin E (Sc-247), c-Myc 9E10 (sc-40), and HA Y-11 (sc-805) were from Santa Cruz Biotechnology. Fbw7 (A301-720A) was from Bethyl Laboratories. α-Tubulin (T-5168), Vinculin (V-4505), polyclonal FLAG (F-2425), monoclonal FLAG (F-3165), HA agarose beads (A-2095), peroxidase-conjugated α-mouse secondary antibody (A-4416) and peroxidase-conjugated α-rabbit secondary antibody (A-4914) were from Sigma. GFP (632380) was from Invitrogen. DAB2IP antibody was previously described [16].

### Immunoprecipitation and Western Blotting

Cells were lysed in EBC-lysis buffer (50 mM Tris, pH 8.0, 120 mM NaCl, and 0.5% NP-40) supplemented with protease inhibitors (Complete Mini; Roche) and phosphatase inhibitors (phosphatase inhibitor cocktail set I and II; EMD Millipore). The protein concentrations of the lysates were measured using a protein assay reagent (Bio-Rad Laboratories, CA) on a DU-800 spectrophotometer (Beckman Coulter). The lysate samples were then resolved by SDS-PAGE and immunoblotted with the indicated antibodies. For immunoprecipitation assays, 20 hrs of post transfection, cells were treated with 10 μM MG132 overnight before harvesting for immunoprecipitation. 1 mg of protein lysates were incubated with the appropriate antibodies (1-2 μg) overnight at 4°C, followed by addition of carrier beads. Immunocomplexes were washed five times with NETN buffer (20 mM Tris, pH 8.0, 100 mM NaCl, 1 mM EDTA, and 0.5% NP-40) before being resolved by SDS-PAGE and immunoblotted with indicated antibodies.
